# Aerosol formation during foam application of non-volatile biocidal substances

**DOI:** 10.1093/annweh/wxad031

**Published:** 2023-06-26

**Authors:** Katharina Schwarz, Katharina Blümlein, Thomas Göen, Stefan Hahn, Ralph Hebisch, Wolfgang Koch, Ulrich Poppek, Anja Schäferhenrich, Urs Schlüter, Monika Krug

**Affiliations:** Fraunhofer Institute for Toxicology and Experimental Medicine ITEM, Nikolai-Fuchs-Str., 30625 Hannover, Germany; Fraunhofer Institute for Toxicology and Experimental Medicine ITEM, Nikolai-Fuchs-Str., 30625 Hannover, Germany; Institute and Outpatient Clinic of Occupational, Social and Environmental Medicine (IPASUM), Friedrich-Alexander-Universität Erlangen-Nürnberg, Henkestr. 9–11, 91054 Erlangen, Germany; Fraunhofer Institute for Toxicology and Experimental Medicine ITEM, Nikolai-Fuchs-Str., 30625 Hannover, Germany; Federal Institute for Occupational Safety and Health, Friedrich-Henkel-Weg 1–25, 44149 Dortmund, Germany; Fraunhofer Institute for Toxicology and Experimental Medicine ITEM, Nikolai-Fuchs-Str., 30625 Hannover, Germany; Federal Institute for Occupational Safety and Health, Friedrich-Henkel-Weg 1–25, 44149 Dortmund, Germany; Institute and Outpatient Clinic of Occupational, Social and Environmental Medicine (IPASUM), Friedrich-Alexander-Universität Erlangen-Nürnberg, Henkestr. 9–11, 91054 Erlangen, Germany; Federal Institute for Occupational Safety and Health, Friedrich-Henkel-Weg 1–25, 44149 Dortmund, Germany; Federal Institute for Occupational Safety and Health, Friedrich-Henkel-Weg 1–25, 44149 Dortmund, Germany

**Keywords:** aerosol formation, biocidal foams, exposure concentration

## Abstract

The application of biocidal products by foam is considered an alternative to droplet spraying when disinfecting surfaces or fighting infestations. Inhalation exposure to aerosols containing the biocidal substances cannot be ruled out during foaming. In contrast to droplet spraying, very little is known about aerosol source strength during foaming. In this study, the formation of inhalable aerosols was quantified according to the aerosol release fractions of the active substance. The aerosol release fraction is defined as the mass of active substance transferred into inhalable airborne particles during foaming, normalised to the total amount of active substance released through the foam nozzle. Aerosol release fractions were measured in control chamber experiments where common foaming technologies were operated according to their typical conditions of use. These investigations include foams generated mechanically by actively mixing air with a foaming liquid as well as systems that use a blowing agent for foam formation. The values of the aerosol release fraction ranged from 3.4 × 10^−6^ to 5.7 × 10^−3^ (average values). For foaming processes based on mixing air and the foaming liquid, the release fractions could be correlated to the process and foam parameters such as foam exit velocity, nozzle dimensions, and foam expansion ratio.

**What’s important about this paper?**

Disinfection of surfaces and application of biocides by foam techniques are considered as an alternative to droplet spraying as foaming is assumed to emit less aerosol. This study quantified the formation of inhalable aerosol as the release fraction of the active substance. For foaming processes based on mixing air with the foaming liquid, a correlation was established relating the release fraction to process and foam parameters. The presented approach enables the assessment of exposure to inhaled active substances, which can be considered when selecting foam technology.

## 1. Introduction

At workplaces, disinfectants and insecticides are usually applied by brushing, soaking, spraying, or foaming (ECHA 2015). Spraying and foaming are primarily used for disinfecting large surfaces. Foaming is a viable alternative to droplet spraying from the perspective of disinfection efficiency (Chlibek et al. 2006; Le Toquin et al. 2018; White et al. 2018). Exposure to airborne active substances during foaming cannot be ruled out and needs to be assessed in the context of risk assessment. For non-volatile active substances such as pyrethroids or quarternary ammonium compounds (QACs), inhalation exposure will be in the form of aerosols. In contrast to spraying (Lorenz et al. 2011; Nazarenko et al. 2014; Schwarz and Koch 2017; Meesters et al. 2018), only limited information exists on the generation of inhalable aerosols during foaming (Schwarz et al. 2015; Ehnes et al. 2019).

Aqueous disinfection foams are mainly generated by mixing air with the liquid biocidal product or its application solution at a desired ratio prior to its release through a nozzle. The foam mixture is produced either actively via the injection of pressurised air into the liquid formulation or is based on the Venturi principle to mix water, a concentrated disinfection solution, and air. Non-aqueous biocidal foams, on the other hand, are generated from pressurised cans (such as rodenticides or insecticides) that contain the disinfection formulation and a blowing agent, for example, a compressed hydrocarbon forming the gas phase of the foam by expansion.

An important parameter characterising foam is the so-called expansion ratio (the inverse of the liquid volume fraction), defined as the ratio of the volumes of foam and liquid (Laundess et al. 2011).

Inhalation exposure to non-volatile active substances during foam application is related amongst others to the source strength describing aerosolised active substance release into the air during foaming. This source strength is defined as the flux of aerosolised active substance released into the air during foaming normalised to the total mass flux of active substance. The proportionality factor is called the aerosol release fraction (Schwarz and Koch 2017) and is an inherent property characterising the foaming technique regarding its potency to form inhalable particles containing the active substance.

This paper deals with the measurement and parameterisation of release fractions for common foaming devices according to their typical conditions of use. We focus on foams generated mechanically by mixing air with a foaming liquid but also provide some exemplary data on systems using a blowing agent.

## 2. Materials and methods

This section details the foaming devices and investigated formulations, the definition of the release fraction, and the outline of the general measurement procedure. Thereafter, the experimental set-up and analytical methods are described.

### 2.1. Foaming technologies

**Fig. 1.**
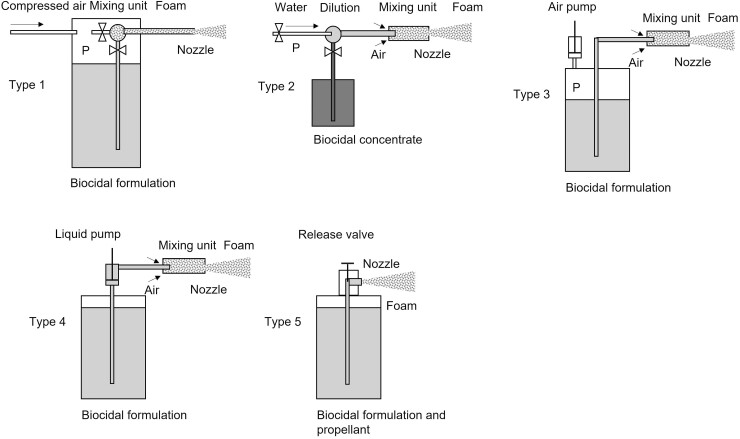
Principles of foam generation of the devices used in this study. Type 1: internal mixing of air and the final formulation. Type 2, 3, and 4: foam generation in a Venturi nozzle. Type 5: foam generation by evaporation/expansion of propellant.

The following commercial foam devices were investigated (see Fig. 1 and Table 1):

- Systems in which air and the biocide formulation were mixed inside a mixing unit to form the foam which was subsequently released through a nozzle (type 1): Two stationary pressure foamers G and B for large-scale foam applications were investigated. In addition, we selected a portable pressure foamer P (carried by the user) for medium- and small-scale applications. All three devices were equipped with a reservoir connected to a compressed-air tube for pressure and air supply. Operating pressures were between 3 and 6 bar. The stationary pressure foamers are regarded as representative of all large-scale stationary installations connected to a water and compressed-air tube and use the same principle of foam generation.
- Devices operated according to the Venturi principle, where the mixing of air and liquid takes place inside a nozzle (type 2): Systems used here included a low-pressure foam gun connected to the in-house water system (water pressures < 6 bar) and a high-pressure foam gun operated by a high-pressure booster (>10 bar). All systems used a combination of two Venturi nozzles: water supplied by a low- or high-pressure pump exited a nozzle at high speed, causing the concentrated foaming agent and air to be sucked into a foam gun. Here, the concentrated foaming agent is ­diluted at the first nozzle at a given ratio (adjustable), and the foam is generated at the second nozzle by introducing air into the diluted formulation. These devices are intended for large-scale applications.
- Hand-compression foamer as a small-scale Venturi-type device (type 3): A device was chosen in which the liquid formulation was pressed out of a reservoir by inflating the reservoir with a hand pump. The pressure started at around 3 bar and declined during operation as the reservoir was emptied. Hand-compression foamers are intended for small-scale applications.
- Hand-pump foamer operated discontinuously and without a defined pressure (type 4): The foaming liquid was intermittently pumped out of a reservoir by a trigger pump and mixed with air in the foam nozzle. Hand-pump foamers are intended for small-scale applications.
- Pressurised cans (type 5): Propellant gases are used for foam formation (here: insect foams) and are intended for small-scale applications.

**Table 1.**
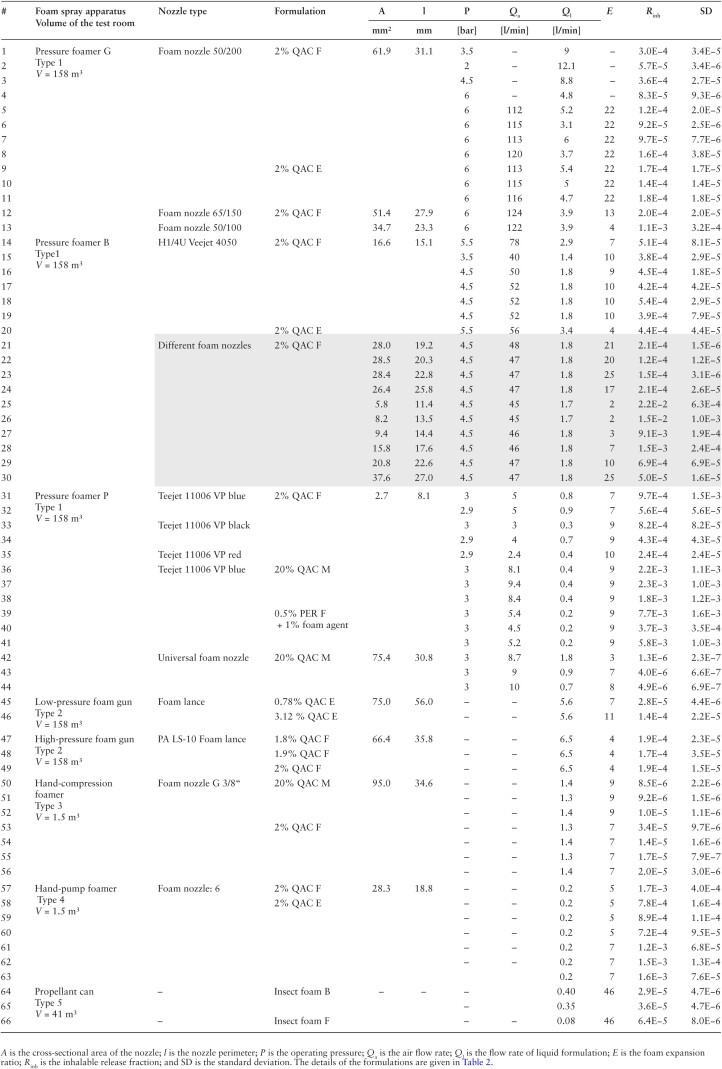
Foaming of disinfectants and insecticides; data on process parameters and inhalable release fractions of the active substance. The data of the shaded rows correspond to measurements carried out with nozzles that are not used with this device in the field.

All continuously operating devices (except the foam cans) could be equipped with different foam nozzles. For type 1 and type 2 devices, the nozzles were either ovaloid or circular in shape with cross-sectional areas between 2.7 and 95 mm², nozzle diameters between 1 and 11 mm (small diameter), and aspect ratios between 1 (circular nozzle) and 3.8. One nozzle was rectangular with a dimension of 25 × 3 mm².

For the devices equipped with a reservoir, the flow rate of the formulation was measured by weighing the reservoir before and after the test. The water flow rate for the pressure foam guns (type 2) was determined by flow metres. The liquid mass flow rate covered a range from 0.2 to 6.5 l/min. Airflow rates for pressure foamers type 1 were measured by a mass flow metre inserted in the compressed-air tube. The volumetric flow rates of the mixing air ranged from 2.4 to 124 l/min. For the type 2 foamers (Venturi principle), the air flow rate could not be directly measured.

The pressure values referred to system operating pressures and did not represent the pressure at the foam nozzle responsible for foam dispersion. The pressure foamer G required two interrelated pressure adjustments, one for the built-in air pressure-driven pump for liquid formulation and a second one for the air supply for foam generation. The latter is listed in Table 1. Recommended pressure values were 5.5 bar for the pump and 6 bar for the air. For the pressure foamer B, the pressure and setting of an internal mixing unit/pressure regulator were the parameters to be adjusted by the user. For the Venturi-type devices, the pressure referred to the water pressure at the first Venturi nozzle.

### 2.2. Formulations

The foam formulations and their compositions are listed in Table 2. The first two products were formulations in propellant aerosol cans. The products containing QACs and the one containing *alpha*-Cypermethrine were concentrated solutions diluted with water prior to application.

**Table 2.** Foam formulations used in the study. With the exception of the foam cans, the products were concentrated and diluted before application. The italicised substances were analysed in the release measurements. The indicated concentrations refer to the final formulation.

Product name	Active substance (content)
Insect foam B	Tetramethrin (*N*-15022: 3.1 g/kg; *N*-72552: 1.5 g/kg), *Phenothrin* *(N-15022*: *1.05 g/kg; N-72552: 1.5 g/kg)*
Insect foam P	Pyrethrine (1 g/l) *PBO (2 g/l)* *Permethrin (28 g/l)*
QAC F	Product Didecyl dimethyl ammonium chloride (33 g/kg) BAC (66 g/kg)
	Test formulation *+ CsCl (0.1%)*
QAC E	Product Didecyl dimethyl ammonium chloride (33 g/kg) BAC (66 g/kg)
	Test formulation *+ CsCl (0.1%)*
QAC M	Product *N*-alkyl(C12-16)-*N*,*N*-dimethyl-*N*-benzyl ammonium chloride (95 g/l)
	Test formulation *+ CsCl (0.1%)*
PER F	Product *alpha*-Cypermethrin (60 g/l)
	Test formulation *+ CsCl (0.1%)*

Caesium chloride was used as a tracer in aqueous-based QAC formulations as a more sensitive analytical method could be employed for its quantification. Hence, for the determination of the inhalable release fraction (see “Definition and measurement of release fractions” section) the aerosol samples were analysed for their CsCl content. Using CsCl as a surrogate for QAC was valid since, in the calculation of the release fraction normalisation was conducted with respect to the measured substance and, therefore, was independent of the substance selected for analysis. For type 1 devices, CsCl was added to the solution in the reservoirs at a final concentration of 0.1% (w/w). For the foam guns (type 2), CsCl was added to the water to dilute the biocidal product concentrate. In these instances, the aqueous CsCl solution (0.1% CsCl) was prepared in the 150 l tank from which the operating pumps were supplied.

### 2.3. Definition and measurement of release fractions

#### 2.3.1. General methodology

The inhalable release fraction of the active substance *R*_inh_ is defined as


$$\begin{array}{l} R_{inh}=\frac{m_{inh}}{M_{s}} \end{array}$$ (1)

where *m*_inh_ [kg] is the total mass of the active substance released as inhalable aerosol, and *M*_s_ [kg] is the total mass of the active substance released as foam. The inhalable aerosol size fraction is defined in the EN standard 481 (CEN 1992; Hinds 1999). It is defined as the wind-orientation-averaged mass fraction of total airborne particles, that are inhaled through the mouth or nose.

A mass balance method described by Schwarz and Koch (2017) was employed to measure the release fractions. This method used a control chamber with homogenous air distribution (volume *V* [m³]) where a foam bolus containing a mass *M*_s_ of the active substance was applied onto a surface according to its conditions of use. During foaming, a certain fraction of the active substance became airborne in the fine foam droplets not deposited on the surfaces. This overspray dispersed throughout the chamber volume, and the volatile components of the foam formulation evaporated, resulting in a matured aerosol consisting of the active substance and other non-vaporised compounds. The amount of the inhalable size fraction of the matured aerosol was measured to determine the release fraction.

Under well-stirred conditions, the aerosol was spatially homogeneous, leading to an inhalable concentration *c*_0_ of the active substance generated during the foaming process inside the control volume immediately after application. The aerosolised mass was calculated from:


$$\begin{array}{l} m_{inh}=c_{0}\cdot V \end{array}$$ (2)

The concentration *c*_0_ [kg/m³] was derived from the time-average value $\bar{c}$ [kg/m³], by aerosol sampling over a sufficiently long measuring time *T* [s] (much larger than the duration of foam release) in order to collect enough material for the chemical analysis of the active substance. Aerosol losses as a result of settling and deposition on the ventilator blades were taken into account by assuming an exponential concentration decrease, *c* = *c*_0_Exp(−*t*/*τ*), where, *c*_0_ is the concentration immediately following the foam process and *τ* is the decay time. This initial concentration was related to the measured time-average concentration $\bar{c}$ and the measured decay time τ by:


$$\begin{array}{l} c_{0}=\bar{c}\frac{T/\tau }{1-e^{-T/\tau }}. \end{array}$$ (3)

The release fraction was obtained from the measured time-average concentration and the decay time by combining Equations 1–3.

### 2.4. Experimental setup, procedures, and measurement methods

The time-averaged concentration of the active substance was determined by chemical analysis of filters (mixed cellulose ester (MCE), ANALYT-MTC Meßtechnik GmbH, Müllheim, Germany) using samplers for inhalable aerosols (GSP, ANALYT-MTC Meßtechnik GmbH, Müllheim, Germany) operating at a flow rate of 3.5 l/min. The decay time was extracted from time-resolved recordings of the inhalable aerosol mass concentration by a laser aerosol spectrometer (LAS, Type 1.109, GRIMM Aerosol Technik Ainring GmbH & Co. KG, Ainring, Germany) sampling at 1 l/min.

**Fig. 2.**
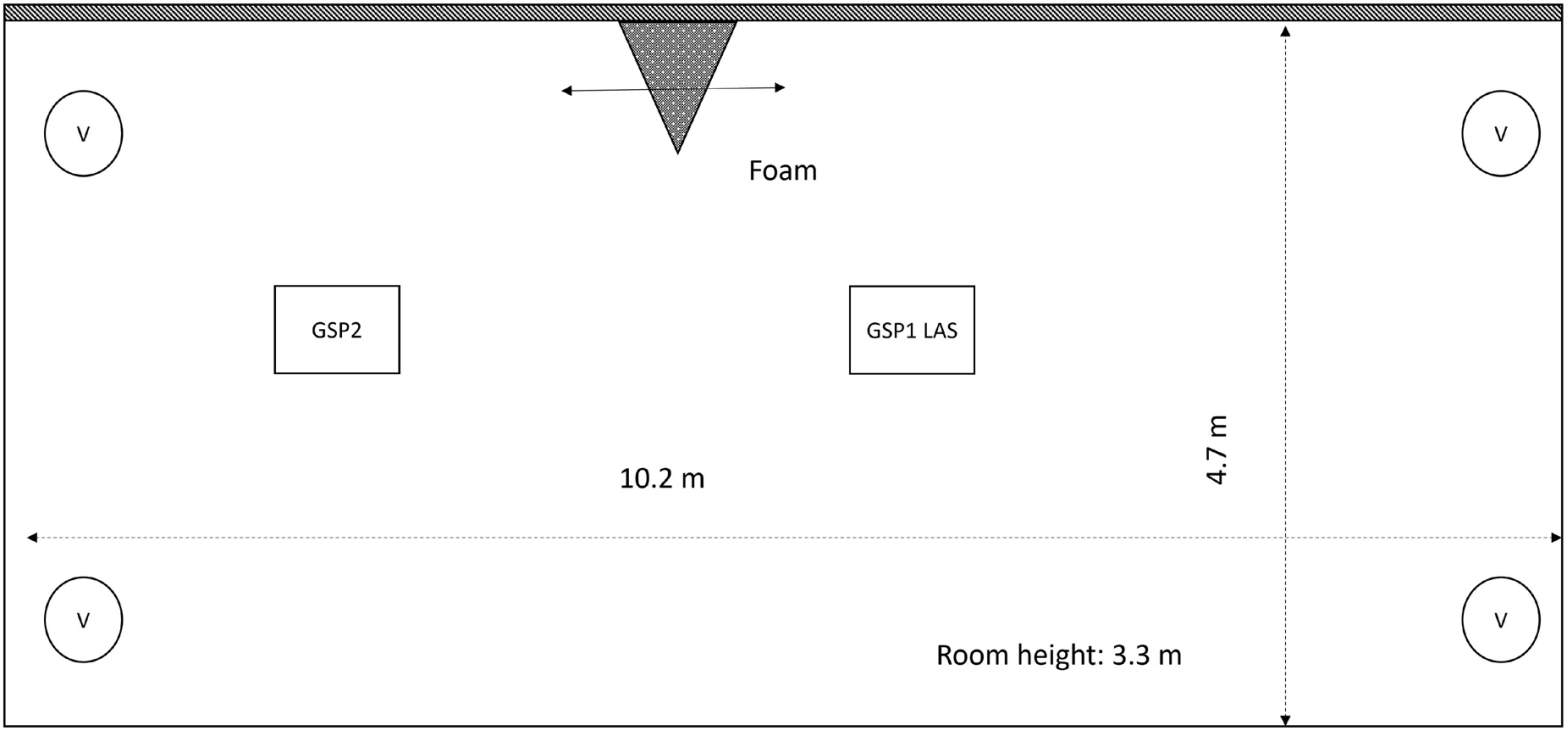
Schematics of the experimental set-up (large control room (158 m^3^)) for the measurement of the release fraction. V: room ventilator, LAS: laser aerosol spectrometer, GSP: sampler for inhalable aerosols.


Depending on the expected aerosol concentration related to the mass release of the different foam systems under consideration, experiments were carried out in control rooms of appropriate size. Three control rooms were available with volumes of either 158 m^3^, 41 m^3^, or 1.5 m³. Two GSP samplers were used at different positions to check for homogeneity of the aerosol concentration. The LAS was placed adjacent to one of the GSP samplers. The experimental set-up in the large control room (158 m³) is shown in Fig. 2. Four commercial room ventilators were operated to generate turbulence for efficient internal air mixing. The external air exchange rate was zero during foam release and aerosol collection. However, there was a small remaining virtual air exchange by the operation of the GSP samplers and the aerosol spectrometer since the aerosol mass sucked in by the instruments was removed from the chamber volume during sampling. One foam layer was applied to the wall as indicated. Overlap of foam layers was avoided during foaming. Foaming durations ranged between 1.5 and 5 min, with sampling times being 10 and 30 min, respectively. At the end of sampling, the sampling pumps were switched off and the room was ventilated with fresh air until the aerosol concentration, monitored with the LAS, was at the background concentration. This procedure was repeated three times, resulting in four release actions. The reproducibility was generally good, so that for some experiments such as the tests with the aerosol cans only one release action was carried out per test. Since the GSP filters were not replaced between foam-release actions, there was only one analytically determined concentration value $\bar{c}$ of the active substance averaged over the foam-release actions. The initial aerosol concentration *c*_0,*i*_ of the individual release actions could vary. This variability was taken into account from the relative heights of the concentration peaks measured by the aerosol spectrometer and is expressed in:

$$\begin{array}{l} c_{0,i}=\bar{c}\cdot \frac{T/\tau }{1-e^{-T/\tau }}\cdot \frac{n\cdot \alpha_{i}}{\overset{n}{\underset{i=1}{\sum }}\alpha_{i}}. \end{array}$$ (4)

The variable *n* represents the number of release actions and the dimensionless quantities $\alpha_{i}=c_{0,i}\;/c_{0,1}$ are the relative heights of the concentration peaks. These factors could be calculated from the data obtained by the aerosol spectrometer because the aerosol mass concentration measured by the aerosol spectrometer is proportional to the mass concentration of the active substance. The aerosol decay time was assumed to be the same for all four foam-release actions carried out during testing with a fixed set of parameters since the experimental conditions controlling the aerosol decay by losses on the walls and the blades of the ventilators as well as losses related to the sampling procedure did not change throughout testing.

The time-averaged concentration $\bar{c}$ of CsCl was determined by analysing the tracer amount on the MCE filters using ICP-MS. The limit of quantification (LOQ) under the experimental procedure was an airborne tracer concentration of 90 ng/m³. In the tests with propellant-driven foam cans containing pyrethroids, the active substance was measured directly using GC-MS with a LOQ of 2.4 µg/m³.

#### 2.4.1. Experimental matrix

A total of 66 release experiments were carried out. In the 158-m^3^ room, 49 tests were done using the large-scale foaming units (type 1 and type 2). In the 1.5-m^3^ chamber, 14 tests were performed with the hand-compression foamer and the hand-pump foamer (type 3 and type 4). The aerosol release of foam cans (type 5) was carried out in the 41 m³ room. Each control room was selected based on the range of the foam jet, the expected release fractions, and the LOQ of the analytical method. Table 1 gives details on the parameters of the foaming devices such as nozzle dimensions, air flow rates, and liquid flow rates. Experiments 1 to 56 were carried out with the commercial devices as delivered and with parameters specified by the supplier, including the recommended operating pressure as well as the recommended foam-nozzle type and any necessary adjustments. Additional experiments (21–30) were carried out using commercial pressure foamer B equipped with nozzles of varying dimensions. This was done to extend the parameter range of process parameters to find correlations between release fractions and process parameters.

## 3. Results and discussion

### 3.1. Method validation

For the aqueous formulations, CsCl was used as a tracer for the measurement of the release fraction. The comparison between tracer and active-substance analysis was carried out in preliminary tests. A foam containing 0.1% CsCl and 0.16% benzalkonium chloride (BAC) was released in the control room (158 m³). The aerosol was collected on filters. Five filter samples were analysed for BAC and CsCl using the methods described above, resulting in a ratio BAC/CsCl of 1.66 ± 0.11 in accordance with the ratio of the contents in the formulation.

**Fig. 3.**
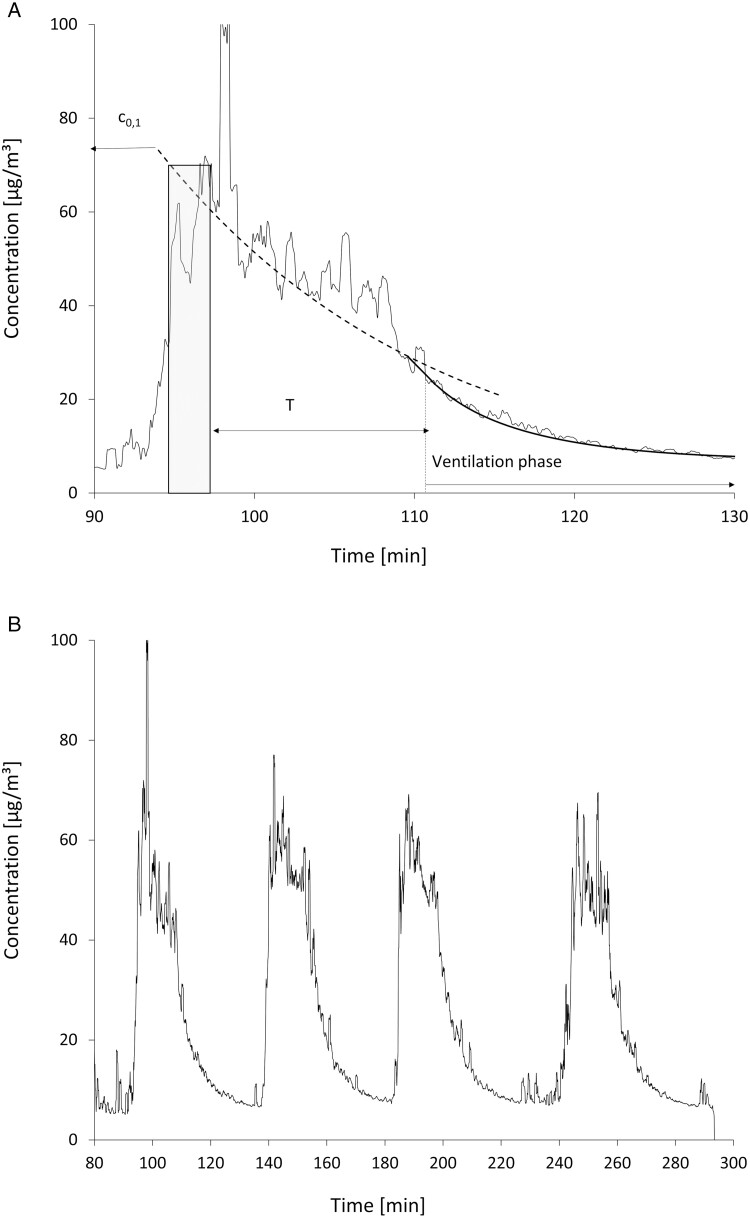
a) The concentration pattern for a release experiment is characterised by an increase during foaming (shaded area) and a decrease due to losses inside the chamber (settling and deposition) that is approximated by an exponential function. A second exponential decrease is caused by the air exchange during the ventilation phase. The area under the curve during T is used to derive the factor connecting time-averaged concentration and the concentration peak, $c_{0,1}$, as the value of the exponential curve approximation at the beginning of the sampling period. b) The time pattern of the aerosol concentration for four foaming actions was carried out consecutively.

The time patterns for an exemplary individual and a 4-fold consecutive foam application as measured with the aerosol spectrometer are shown in Fig. 3a and b. The individual pattern is characterised by a short foaming period (shaded rectangle in Fig. 3a), a longer residence period of the aerosol with zero ventilation and a ventilation phase for removing airborne particles before the next foam application. Aerosol sampling was carried out during the first two time periods. The pattern during sampling was approximated by an exponential curve from which the decay time τ was determined (dashed line; $c_{0,1}=69$ mg/m³, τ=17 min, T =16 min). This leads to a value of 1.44 for the correction factor $T/\tau /(1-e^{-T/\tau })$, relating the analytically determined average concentration $\bar{c}$ and the peak concentration used for calculating the release fraction. An error in the decay-time determination of ±20% causes an error of ±7% in the correction factor. This was accepted and the decay time was determined from only one of the four release actions.

**Fig. 4.**
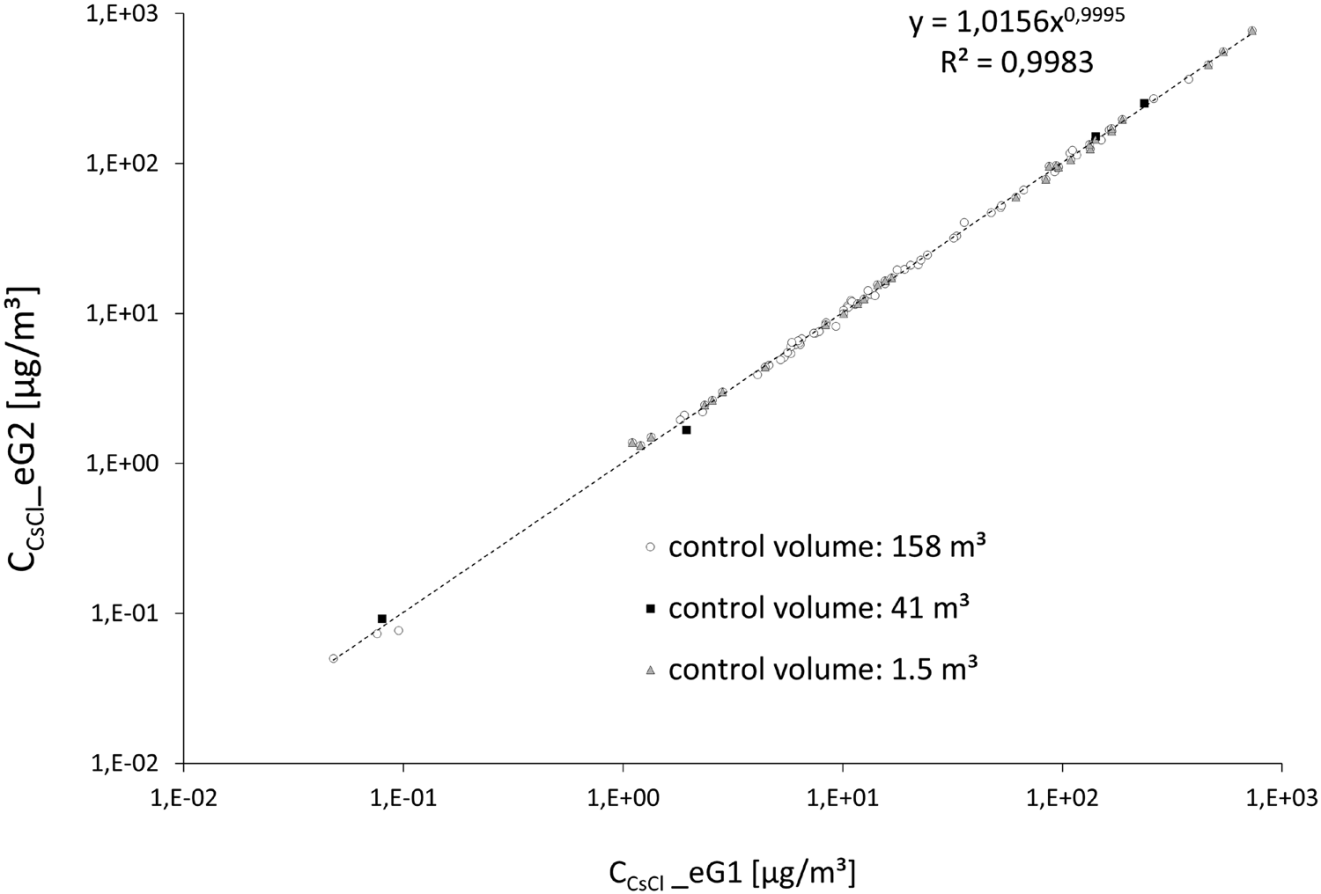
The analytically determined, time-averaged concentration of the active substance or tracer obtained for all release measurements from the samples collected with GSP1 and GSP2 are identical and cover four orders of magnitude.

Another prerequisite for determining the release fraction from concentration measurements is the spatial aerosol homogeneity inside the control volume. This was fulfilled for all three experimental settings: the concentration data obtained from the two GSP samplers show perfect agreement (Fig. 4): $c_{GSP2}=1.0156\cdot c_{GSP1}^{0.9995},\;R^{2}=0.9983$.

### 3.2. Data on aerosol release fraction

**Fig. 5.**
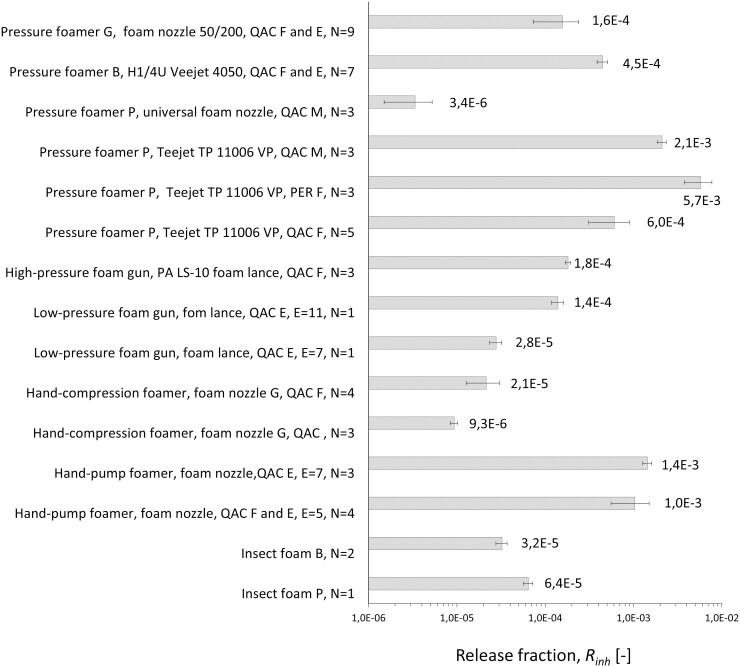
Inhalable release fractions of non-volatile disinfectant and pyrethroid, respectively measured for a selection of commercial foam-generation devices. The underlying numerical data and the related process parameters are given in Table 1.

The results of all measurements are listed in Table 1. The average value of the inhalable release fraction of the active substance is shown in column *R*_inh_ and the standard deviation resulting from the repeated (2–4) foam applications carried out during a test under identical experimental parameters is given in column SD. The data were sorted with respect to the device (second column), the foam nozzle used (third column), and the formulation used in the test (fourth column). Fig. 5 shows average release fractions obtained in 52 experiments, grouped by devices and nozzles typically used in the field. For this figure data obtained with the foam formulations QAC F and QAC E were combined as these formulations have very similar compositions. Fig. 5 gives an overview of the range of release fractions for each device and the formulation used, and their variance when operated under real workplace conditions as the experimental test parameters represented field conditions in view of the mode of application, nozzle types typically used, and the operating parameters of the device. The error bars represent the standard deviation of the average release fractions in the underlying data set containing N  values. For the low-pressure foam gun and the foam can containing the insect foam P, only one test was carried out (*N* = 1) and the standard deviation was based on the results of the repeated foam applications within the setting.

The aerosol formation potential of the various devices covered more than three orders of magnitude. At the upper end, up to 0.6% of the non-volatile active substance was released as inhalable aerosol during foam application. At the lower end, only a few ppm of the foamed active substance were transformed into airborne inhalable particles. The two extremes were obtained using the same foam-generation device (pressure foamer P) but quite different cross-sectional areas of the foam nozzles (75.4 mm² (universal foam nozzle) versus 2.75 mm² (Teejet 11006 VP)) and, in turn, a large difference in the exit velocity of the foam: 2.2 m/s for the large nozzle and 34 m/s for the small nozzle. Shear forces acting on the surface of the foam jet upon exiting the nozzle were assumed to be primarily responsible for the generation of small foam droplets. This suggested a correlation of the release fraction *R*_inh_ with the factor $v\cdot l^{-1}$ where v is the exit velocity of the foam through the foam nozzle, calculated from the total flow rate of air and liquid ($Q_{a}+Q_{l}$) and the cross sectional area A of the nozzle: $v=\left(Q_{a}+Q_{l}\right)/A$. The length l is the nozzle circumference. The smaller the nozzle dimensions, the larger is the specific surface area (surface area per unit volume) from which airborne droplets are assumed to be generated. Furthermore, the release fraction, *R*_inh_, of active substance was expected to correlate with the inverse of the foam expansion ratio, E (>1). The smaller the foam expansion ratio (foam volume/volume of the liquid formulation containing the active substance), the higher is the amount of the active substance in the airborne foam particles generated at the nozzle at constant flow rate, $Q_{l}$, of the liquid formulation through the nozzle. The limiting situation is that of a sprayer, where E=1.

**Fig. 6:**
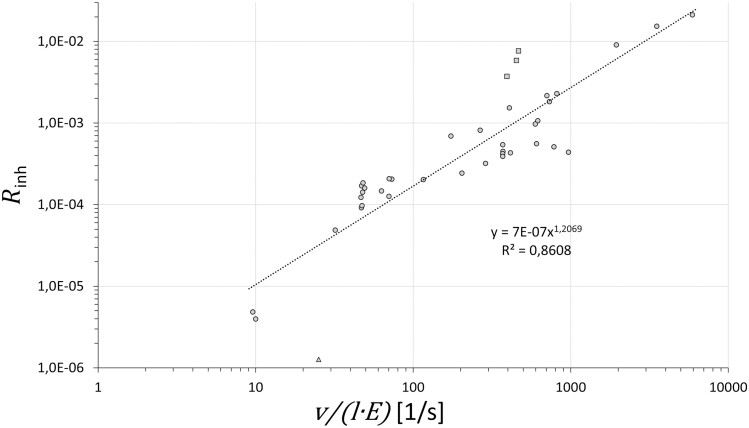
The relationship between the process parameters exit velocity, v, nozzle perimeter length, l, foam expansion ratio, E_,_ and the release fraction, $R_{inh}$ of the inhalable aerosol of the active substance. Data are based on experiments carried out with type 1 devices where liquid and air flow rates were directly measured. Triangle: possible outlier, rectangles: special formulation using foaming agent (see text).

Therefore, the release fraction *R*_inh_ was assumed to correlate with the process variables according to v/(E⋅l). The applicability of this assumption was tested using the data obtained in the experiments carried out with type 1 devices that used active, internal foam mixing. Here, the flow rate of the foam air $Q_{a}$ and the liquid flow rate $Q_{l}$ were measured in order to calculate the foam-exit velocity. To extend the database, additional experiments were carried out with the pressure foamer B device equipped with different foam nozzles (21–30 in Table 1). The data supported this correlation as the relationship between $R_{inh}$ and v/(E⋅l) was almost linear (Fig. 6). The triangle belonged to experiment 42, one of the three experiments carried out with the universal foam nozzle (*A* = 75.4 mm²). This was probably an outlier related to measurement errors since the release value was close to the LOQ and the value of the expansion ratio was significantly different from the values of the other two repetitions (43 and 44). The rectangles represent tests 39–41, which were carried out with the PER F formulation that needed a foaming agent to produce foams in the pressure foamer P device. The data may be subject to error since the formulation was not a clear solution but a suspension, and a sediment remained in the reservoir at the end of the test. If these data were removed from the list, the exponent in the regression reduces from 1.21 to 1.08. These results suggested that the dominating mechanism was aerosol formation from the boundary of the foam stream exiting the nozzle. Aerosols stemming from splashing when the foam impacts the surface seemed to have a minor contribution. Aerosol formation related to drainage of the foam layer and the break-up of foam bubbles has not been investigated in this study.

The correlation can be used to estimate the aerosol formation potency from operating parameters and foam properties.

## 4. Conclusions

Foaming is always associated with the generation of inhalable aerosols. The parameters which influence the aerosol release fraction of active substances were derived from systematic control chamber measurements. The active substance release fraction was shown to be a unique indicator of the aerosol formation potency of the technology (device-nozzle combination) under consideration. Values of the release fraction varied by almost three orders of magnitude ranging from 1 to 1,000 ppm. As a basic principle, high foam exit-velocities from small nozzles should be avoided to minimise exposure, because such parameters resulted in the highest values of release fraction. The nominal operating pressure of the foam technology appeared to be a less useful parameter for determining aerosol formation. In contrast, we have identified processes at the foam nozzle, such as foam expansion ratio and exit velocity, which—depending on nozzle-size geometry—control aerosol formation. Except for a preliminary investigation (Schwarz et al. 2015), there are no other equivalent studies in the literature on aerosol formation during foaming. As pointed out in the introduction, there are several investigations on release fractions for spray applications. In Schwarz et al. (2015) a direct comparison was carried out with the results that a pressure foamer type P spray application leads to (max) three-fold higher release fractions as compared to foam application. The data on the release fraction are useful for deterministic exposure modelling as they determine the source term in dispersion models.

## Data Availability

Full data can be found in the report “F2366-Human exposure to biocidal products: Measurement of inhalation and dermal exposure during the application of biocide foams” (https://www.baua.de/EN/Service/Publications/Report/F2366-2.html).
